# Case report: *Mycobacterium monacense* isolated from the blood culture of a patient with pulmonary infection

**DOI:** 10.1186/s12879-020-4936-9

**Published:** 2020-03-12

**Authors:** Chenyan Yuan, Huixia Lu, Congshan Yang, Wei Gao, Hailiang Wang, Guoqiu Wu

**Affiliations:** 1 Department of Clinical Laboratory, Affiliated Zhongda Hospital, Southeast University, Nanjing, China; 2 Department of Critical Care Medicine, Affiliated Zhongda Hospital, Southeast University, Nanjing, China

**Keywords:** *Mycobacterium monacense*, Infection, Blood stream, Severe pneumonia

## Abstract

**Background:**

The poorly known mycobacterial species *Mycobacterium monacense* is a rapidly growing non-tuberculous mycobacterium that was first described in 2006 (Reischl et al., Int J Syst Evol Microbiol 56:2575-8, 2006); it has been reported that its isolation is usually associated with skin and lung infections, especially in immunosuppressed patients (Hogardt et al., Jpn J Infect Dis 61:77-8, 2008; Taieb et al., J Hand Surg Am 33:94-6, 2008; Therese et al., Lung India 28:124-6, 2011; Shojaei et al., Ann Lab Med 32:87-90, 2012; Romero et al., New Microbes New Infect 10:112-5, 2016 ). The clinical significance of *Mycobacterium monacense* is not yet fully understood. Here, we report the first isolation of *Mycobacterium monacense* from the blood culture of a patient in China with severe pneumonia.

**Case presentation:**

On June 26, 2018, a 38-year-old man was admitted to the intensive care unit with breathing difficulty. One day prior, he was discovered with his face immersed in a small pond (non-chlorinated water) and with limb convulsions. He had undergone craniocerebral surgery after trauma 5 years earlier, which left him with epilepsy as a sequela. Bilateral diffuse ground-glass opacity was found in the lungs on chest X ray and chest CT image at admission. The result of the HIV serology test of the patient was negative. The patient was diagnosed with severe pneumonia. Drug-susceptible *Klebsiella pneumoniae* and *Candida glabrata* were isolated in the BALF, and yellow-pigmented colonies were isolated from blood cultures of the patient. The strain isolated from blood was identified by 16S rDNA sequencing as *Mycobacteria monacense*, which is a rapidly growing mycobacterium (RGM). The patient was treated with a combination of cefoperazone sulbactam, linezolid and voriconazole for 10 days, and the symptoms improved. During the one-year follow-up time, the patient did not relapse.

**Conclusions:**

We report the first case of *M. monacense* isolated from blood cultures in a patient with severe pneumonia, which provided evidence that the environmental microorganism possessed pathogenic characteristics.

## Background

From a global clinical perspective, the *Mycobacterium tuberculosis* complex (MTBC) is the most important group within the genus *Mycobacterium* and includes the majority of significant human mycobacterial pathogens [1]. Non-tuberculous mycobacteria (NTM), also known as atypical mycobacteria, are considered widely distributed environmental organisms [2]. NTM are opportunistic pathogens, and the diseases that they cause are related to host factors such as age, immune compromise, chronic diseases and other conditions along with exposure. Different species of NTM can cause pulmonary infection, skin and soft tissue infection, bone and joint infection, lymphatic inflammation and catheter-related bacteraemia [3–5]. *Mycobacterium monacense* is a rapidly growing mycobacterium (RGM) that was first described in 2006; since then, several studies have reported the isolation of *M. monacense* from human clinical samples, such as skin, sputum, and soft tissues [6–11]. Here, we report the first case of *Mycobacterium monacense* isolated from the blood culture of a patient with severe pneumonia.

## Case presentation

**Fig. 1:**
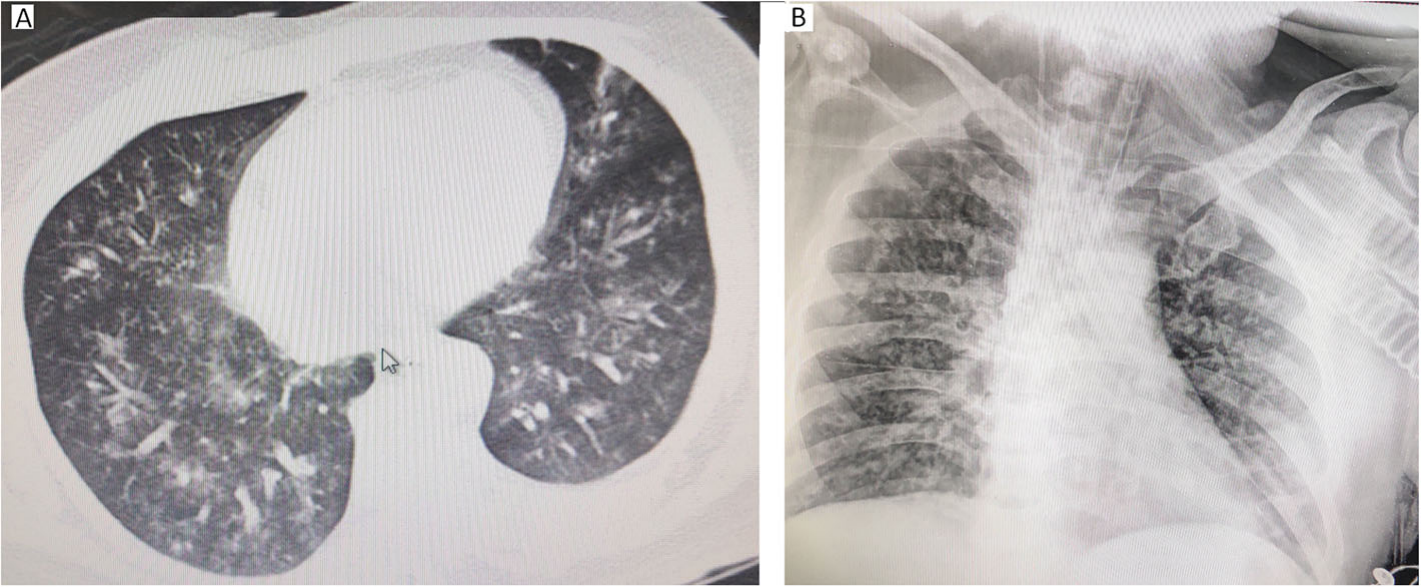
**a** Image of Chest CT scan and **b** Chest radiograph taken at admission

A 38-year-old man was admitted to the intensive care unit (ICU) of our hospital on June 26, 2018 for breathing difficulty. The patient had a five-year history of epilepsy and usually took oxcarbazepine and topiramate to control symptoms. One day prior, the patient was found unconscious with his face immersed in a small pond and with limb convulsions. Ten minutes later, he was rescued and sent to a basic hospital. The patient’s urine volume was decreased, and no improvement was found in dyspnoea after orotracheal intubation. Then, he was sent to our hospital for further treatment. On physical examination at admission, the pulse was 100 beats per minute, the blood pressure was 142/70 mmHg, the temperature was 36.7 °C, and the oxygen saturation was 98% with mechanical ventilation. Laboratory evaluation showed a leucocyte count of 23.22 × 10^9^/L (reference range: 3.5–9.5 × 10^9^/L) with 88.4% neutrophils (reference range: 45–70%), a C-reactive protein level of 313.04 mg/L (reference range: 0–10 mg/L), and a negative result of an HIV serology test. The PaO_2_/FiO_2_ was 259.1 mmHg. A chest radiograph showed diffuse ground-glass opacity in both lungs (Fig. 1). The head CT showed bilateral frontotemporal lobe softening and hydrocephalus, which were sequelae of craniocerebral trauma surgery 5 years prior. The patient was diagnosed with severe pneumonia complicated by acute respiratory distress syndrome (ARDS), epilepsy, and acute kidney injury (AKI). Bronchoalveolar lavage and sputum aspiration were performed immediately at the bedside, and bronchoalveolar lavage fluid (BALF) was sent for culture and metagenomic next-generation sequencing (mNGS). A blood culture and G test for beta-D-glucan were performed at the same time. The patient received continuous renal replacement therapy (CRRT) and was treated with imipenem intravenously as empirical anti-infection therapy.

**Fig. 2:**
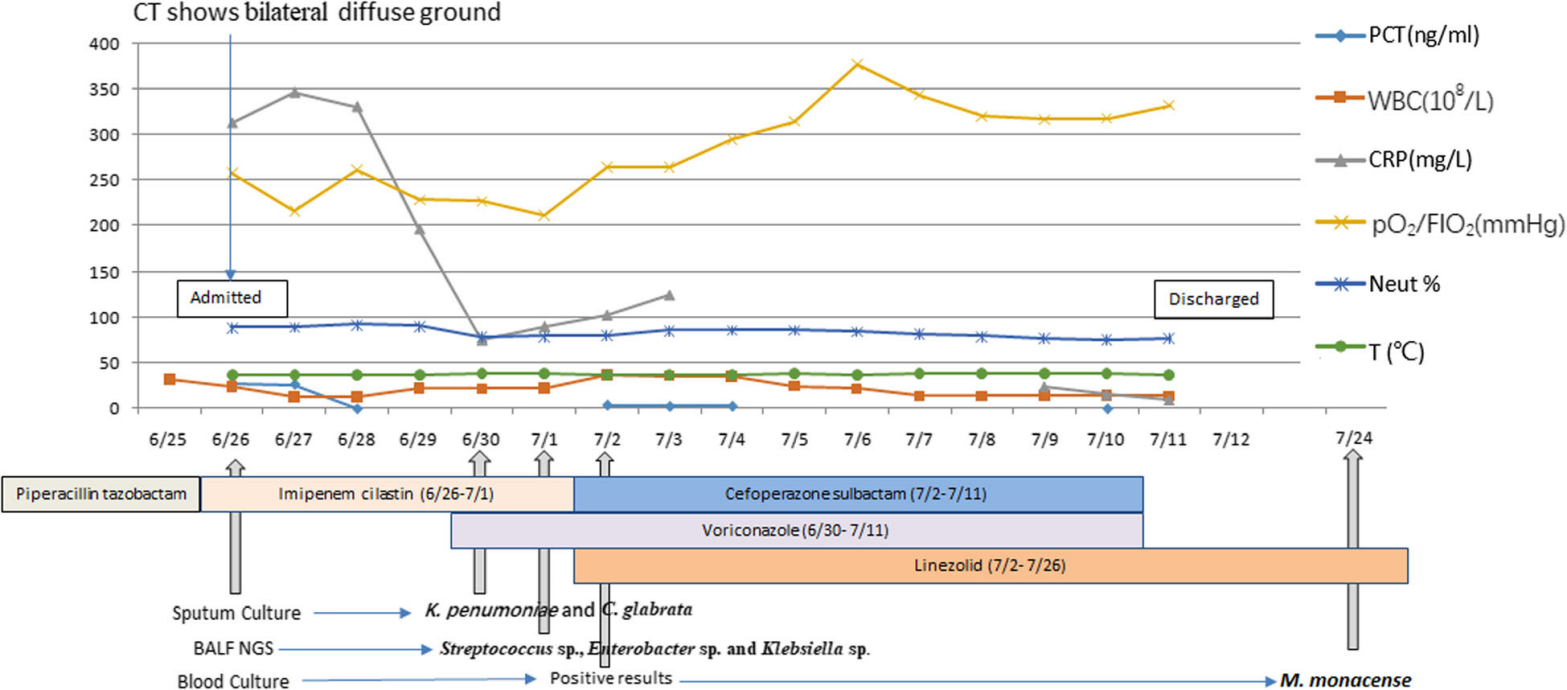
Antimicrobial regimen history, symptoms and laboratory data

On June 30, 2018, drug-susceptible *Klebsiella pneumoniae* and *Candida glabrata* were isolated in the BALF. The value of the G test was less than 10 pg/ml. The results of BALF NGS were returned on July 1, 2018 and showed the presence of *Streptococcus* sp., *Enterobacter* sp. and *Klebsiella* sp., but no fungi or viruses were found. On July 2, 2018, the blood culture of two bottles from different limbs all showed positive growth of “gram-positive cocci”. Based on the results of NGS, BALF culture, and blood culture, the patient’s treatment was changed to combination therapy with cefoperazone sulbactam, linezolid and voriconazole. After 10 days of triple antibiotic combination therapy, the patient’s general condition improved. His blood and sputum culture were negative, renal function recovered, and severe pneumonia improved. Afterwards, the patient was discharged from the hospital and returned to a local hospital for continuing treatment on July 12, 2018. The antimicrobial regimen history, symptoms and laboratory data are shown in Fig. 2.

**Fig. 3:**
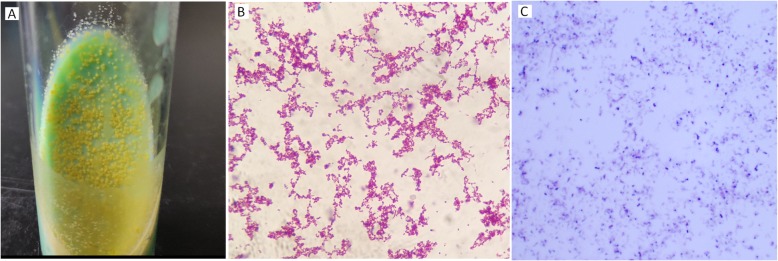
**a**. Colonies of the strain on Lowenstein-Jensen medium. **b**. Acid-fast staining of the strain isolated from blood culture. **c**. Gram staining of the strain isolated from blood culture

**Fig. 4:**
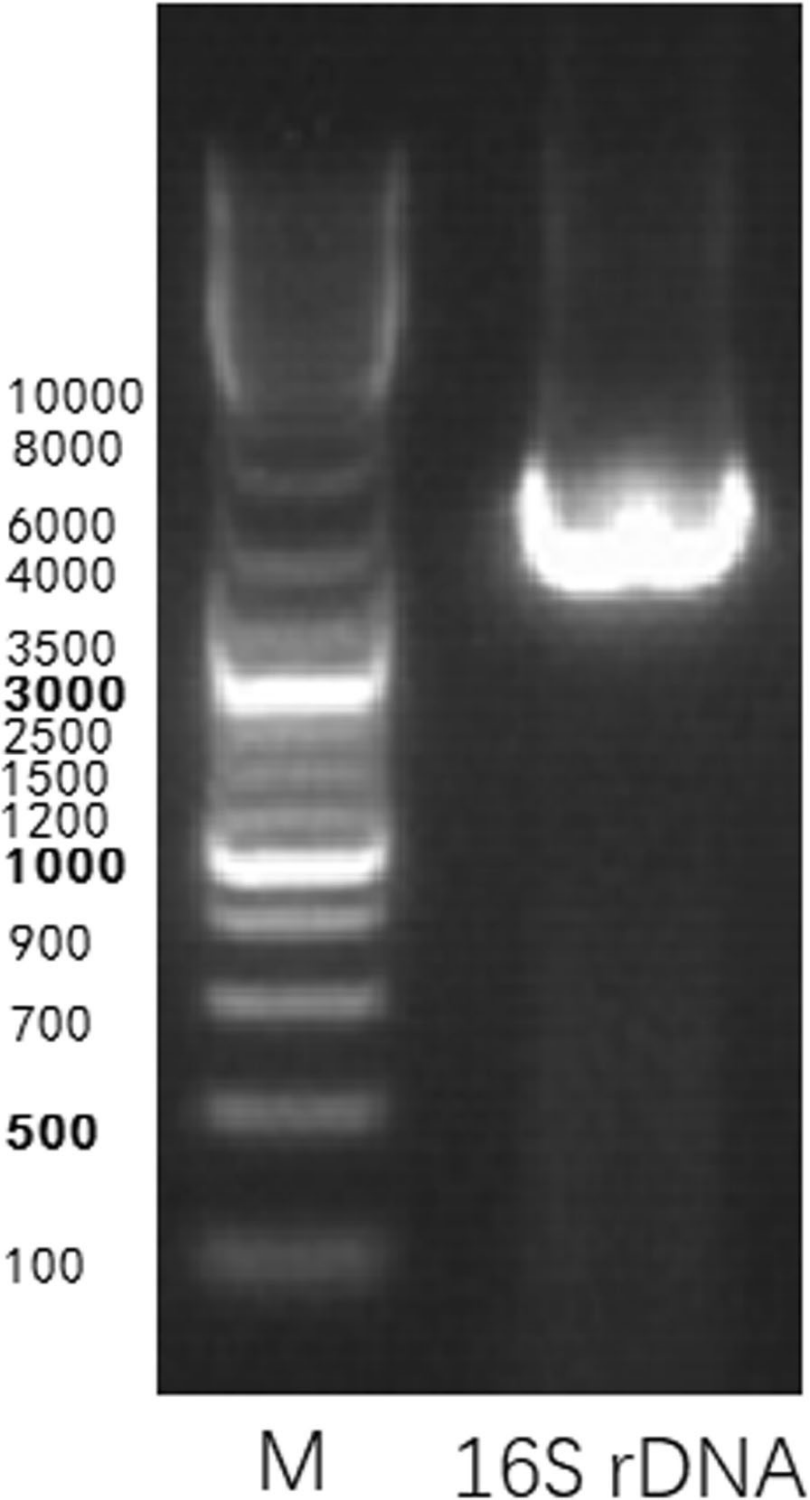
Gel electrophoresis of the 16S rDNA. M: marker

The strain isolated from the patient’s blood showed yellow-pigmented, non-photochromogenic colonies, which took 5 days to grow on Columbia blood agar. The strain was gram-staining variable and acid-fast staining positive, as shown in Fig. 3. The 16S rDNA was amplified (Fig. 4) and identified by sequencing. The nucleotide sequences were analysed with the National Center for Biotechnology Information BLAST (http://blast.ncbi.nlm.nih.gov). The almost full-length (1474-bp) 16S rDNA gene sequence of the bloodstream isolate shared 100% identity with that of *M. monacense* strain B9–21-178 [6]. Susceptibility testing was performed using the broth microdilution method as recommended [12], showing the following minimal inhibitory concentrations (MICs, g/ml): linezolid 2, ciprofloxacin 0.25, cefoxitin 8, imipenem 2, clarithromycin 2, tobramycin 4, amikacin 1 and moxifloxacin 0.25. All of the above MICs were interpreted as “susceptible” based on the breakpoints listed in the CLSI Document M24-2A [12]. The patient was informed of the diagnosis of *M. monacense* bloodstream infection by phone, and he did not return for further consultation. During the one-year follow-up time, the patient did not relapse.

## Discussion and conclusion

To our knowledge, to date, there are nine cases reported in the literature on the isolation of *M. monacense* associated with human infections, yet the clinical significance of *M. monacense* is not fully understood [6–11]. Among the nine cases, six strains were isolated from respiratory specimens, and the other clinical specimens were three biopsy samples of skin fistula tissue and hand and skin nodular lesions. The clinical relevance of the isolation was unclarified in half of the cases reported. Here, we report the first case of *M. monacense* isolated from the blood culture in a patient with severe pneumonia. Drowning injured the patient’s lung, and the infection ensued. It was supposed that the patient was co-infected with *M. monacense, Klebsiella pneumoniae* or other microorganisms. The isolation of *M. monacense* from blood culture, though it was not detected in BALF NGS and culture, provided evidence that the environmental microorganism possessed pathogenic characteristics. The absence of *M. monacense* in BALF NGS might be attributed to the thick cell wall of the mycobacterium, which is hard to break and extract nucleic acids in routine sequencing tests. Fortunately, the patient underwent more transient bacteraemia than blood dissemination of the bacteria, and the symptoms were controlled after antibiotic treatment. In the subsequent drug susceptibility test, the strain was susceptible to most of the antibiotics recommended, including linezolid and imipenem. Such a case implied that *M. monacense,* similar to other NTM, is widely present in the environment (for example, in water and soil) and occasionally infects humans, causing pathological conditions. The pathogenic mechanism of this poorly known mycobacterial species deserves further study.

## Data Availability

The datasets used and/or analysed during the current study are available from the corresponding author on reasonable request.

## Abbreviations

AKI: Acute kidney injury
ARDS: Acute respiratory distress syndrome
BALF: Bronchoalveolar lavage fluid
CLSI: Clinical and Laboratory Standards Institute
CRP: C-Reactive protein
CRRT: Continuous renal replacement therapy
CT: Computed tomography
DNA: Deoxyribonucleic acid
HIV: Human immunodeficiency virus
ICU: Intensive care unit
*M. monacense*: *Mycobacterium monacense*
Neut: Neutrophil
NGS: Next-generation sequencing
NTM: Nontuberculous mycobacteria
PCT: Procalcitonin
RGM: Rapidly growing mycobacterium
T: Temperature
WBC: White blood cell
